# PGC-1α in exercise and fasting-induced regulation of hepatic UPR in mice

**DOI:** 10.1007/s00424-018-2159-3

**Published:** 2018-06-30

**Authors:** Caroline M. Kristensen, Mette A. Olsen, Henrik Jessen, Nina Brandt, Jacob N. Meldgaard, Henriette Pilegaard

**Affiliations:** Department of Biology, University of Copenhagen, August Krogh Building, Universitetsparken 13, 4th floor, Copenhagen, Denmark

**Keywords:** Liver, UPR, Exercise, Fasting, PGC-1α

## Abstract

The aim of the present study was to test the hypothesis that PGC-1α is involved in the regulation of hepatic UPR and autophagy in response to both exercise and fasting in mice. Liver-specific PGC-1α knockout (LKO) mice and their floxed littermates (lox/lox) were used in two experimental parts. Liver and plasma were obtained from (1) fed and 18 h fasted mice and (2) immediately after, 2, 6, and 10 h after 1-h treadmill running as well as from resting mice, where one resting group was euthanized at time points corresponding to 0 and 2 h and another corresponding to 6 and 10 h of recovery. Hepatic eIF2α phosphorylation and sXBP1 mRNA content increased immediately after exercise and IRE1α phosphorylation as well as cleaved ATF6 protein content was higher 2 h into recovery than at rest in both genotypes. Fasting reduced hepatic IRE1α phosphorylation and protein content as well as PERK protein and sXBP1 mRNA content similarly in lox/lox and LKO mice. In addition, the hepatic LC3II/LC3I protein ratio increased immediately after exercise and with fasting in both genotypes, while fasting decreased p62 protein content in lox/lox mice. Liver-specific PGC-1α knockout did not affect these responses, but the LC3II/LC3I protein ratio was higher in LKO than lox/lox mice in both rest groups. In conclusion, the present study provides evidence for pathway-specific exercise-induced activation and fasting-induced downregulation of the UPR as well as exercise and fasting-induced regulation of autophagy in mouse liver. In addition, overall PGC-1α does not seem to be required for the fasting and exercise-induced regulation of UPR and autophagy, but may be involved in regulating basal hepatic autophagy.

## Introduction

Prolonged exercise and fasting impose metabolic challenges to multiple organs of the body including the liver. This leads to acute metabolic regulation with glucose production in the liver through enhanced hepatic glycogenolysis and gluconeogenesis in an attempt to maintain glucose homeostasis [8, 39]. Accordingly, hepatic phosphoenolpyruvate carboxykinase (PEPCK) and glucose-6-phosphatase (G6Pase) activity has been shown to increase immediately after exercise in rats [8]. Moreover, hepatic mRNA and/or protein content of PEPCK and G6Pase have been demonstrated to increase during exercise and fasting in rodents [2, 13, 19, 21].

Metabolic challenges have also been shown to be associated with endoplasmic reticulum (ER) stress and autophagy in the liver [6, 10, 12, 17, 36]. ER stress is characterized by an unfolded protein response (UPR), which aims at reestablishing homeostasis within the ER by reducing the protein load as well as increasing folding capacity and degradation of misfolded proteins [29]. These processes are regulated through three ER membrane bound stress sensors, protein kinase-like ER-kinase (PERK), inositol-requiring enzyme 1α (IRE1α), and activating transcription factor 6 (ATF6). During non-stressed conditions, the chaperone binding immunoglobulin protein (BiP) renders these sensors inactive, while accumulation of unfolded proteins in the ER leads to dissociation of the stress sensors from BiP [3]. This results in the translocation of ATF6 to the Golgi, where it is cleaved to an active transcription factor, cleaved ATF6, while PERK and IRE1α dimerize and autophosphorylate. Activated PERK phosphorylates its downstream target, eukaryotic translation initiation factor 2α (eIF2α), resulting in inhibition of general translation [16], whereas phosphorylated IRE1α activates its endoribonuclease activity leading to the splicing of X-box binding protein 1 (XBP1) into the active transcription factor, spliced (s) XBP1 [42]. Together, the three pathways function in concert to reduce protein synthesis, promote expression of chaperones, and increase degradation of misfolded proteins.

Exercise and fasting have been shown to induce hepatic UPR in rodents. Thus, we have recently reported that an acute bout of exercise was associated with increased phosphorylation of PERK indicating activation of the UPR as well as elevation of the capacity of the IRE1α pathway in mouse liver (unpublished, Kristensen et al.). In addition, a transient increase in hepatic heat shock protein 72 (HSP72) and BiP mRNA content has been observed after a treadmill exercise bout in rats [12] and mice (unpublished, Kristensen et al.). Similarly, previous studies have demonstrated increased ATF6 mRNA and activation of the ATF6 pathway with 24 h of fasting [6] as well as increased IRE1α phosphorylation after 6–18 h of fasting in mice [36]. This suggests that the challenge exerted by exercise and fasting necessitates a transient activation of the hepatic UPR in an attempt to regain ER homeostasis in the liver. However, the molecular mechanisms behind exercise and fasting-induced regulation of hepatic UPR remain to be determined.

Autophagy is the process of degrading cytosolic components and an acute bout of exercise has previously been shown to elevate the hepatic protein content of LC3II [17], which is typically used as a marker of autophagosome number [20]. Similarly, the LC3II/LC3I protein ratio, which often is used as a marker of autophagy [25] has been reported to increase transiently in mouse liver immediately after exercise (unpublished, Kristensen et al.). Moreover, LC3II and the LC3II/LC3I ratio have been shown to increase in mouse liver in response to 24 h of fasting and this was suggested to assist in maintaining glucose levels during fasting [10]. However, the molecular mechanisms behind fasting and exercise-induced regulation of autophagy in the liver are unresolved.

The transcriptional coactivator peroxisome proliferator-activated receptor-gamma coactivator 1alpha (PGC-1α) has been suggested to mediate exercise training-induced metabolic adaptations in mouse liver [13] and to be involved in fasting-induced hepatic metabolic responses including gluconeogenesis [18, 41]. Thus, the hepatic PGC-1α mRNA content has been shown to increase immediately after a single bout of exercise [1, 19] and in response to fasting in mice [15, 41]. Furthermore, PGC-1α was demonstrated to be mandatory for the exercise and exercise training-induced increase in hepatic Cyt c and COXI mRNA and protein content, respectively, in mice [13]. In addition, PGC-1α has been suggested to mediate autophagy [14, 37] and UPR regulation in skeletal muscle [40]. PGC-1α is therefore a likely candidate to play a role in exercise and fasting-induced regulation of hepatic UPR and autophagy. However, whether hepatic PGC-1α plays a role in such regulation in the liver remains to be elucidated.

Therefore, the aim of the present study was to test the hypothesis that PGC-1α is required for the regulation of hepatic UPR and autophagy in response to exercise and fasting in mice.

## Materials and methods

### Mice

Liver-specific PGC-1α knockout (LKO) mice used in the present study were generated by crossing mice homozygous for PGC-1α alleles with exon 3 and 5 flanked by loxP sites [11, 30] with mice homozygous for floxed PGC-1α and heterozygous for albumin-cre recombinase [34]. Littermate floxed (lox/lox) mice were used as controls. Initially, mice were genotyped for the floxed PGC-1α allele and the presence or absence of albumin-cre on gDNA isolated from an ear piece using PCR as previously described [24]. After euthanization, liver PGC-1α knockout was confirmed by determining the PGC-1α mRNA level in liver tissue from all mice. Mice were kept on a 12:12-h light/dark cycle (light cycle: 6 a.m.–6 p.m., dark cycle: 6 p.m.–6 a.m.) at 22 °C and received standard rodent chow (Altromin no. 1324; Brogården, Lynge, Denmark) and water ad libitum if not otherwise stated.

Experiments were approved by the Animal Experiment Inspectorate in Denmark and complied with the European convention for the protection of vertebrate animals used for experiments and other scientific purposes (Council of Europe, no. 123 Strasbourg, France, 1985).

### Experimental setup

#### Exercise study

Male LKO and littermate lox/lox mice were housed in groups until the age of 11–13 weeks after which the mice were single-housed for 1 week, while being acclimatized to the treadmill (TSE Mouse Treadmill System, 303,401 Series) by running 10 min twice a day for 5 days. The last acclimatization was performed 24 h before the experimental day. On the day of the experiment, mice performed 1-h treadmill running at 15 m/min and 10° incline executed between 7 and 9 a.m. Mice were euthanized by cervical dislocation immediately after the exercise bout and 2, 6, and 10 h into recovery. Two distinct control groups were included to account for potential effects of circadian rhythm, and these were resting in their cage until euthanization. Thus, resting mice serving as control for the 0 and 2 h mice were euthanized between 8 and 9 a.m. (*n* = 5 of each genotype) or 10–11 a.m. (*n* = 5 of each genotype) (rest a.m.) and resting mice serving as control for the 6 and 10 h mice were euthanized between 2 and 3 p.m. (*n* = 5 of each genotype) or 5–6 p.m. (*n* = 5 of each genotype) (rest p.m.). Food and water were available ad libitum throughout the experiments.

#### Fasting study

Male and female LKO and littermate lox/lox mice were housed in groups until the age of 11–14 weeks after which they were divided into either a fed or a fasting group (male *n* = 3, female *n* = 5 in each group). Food was removed from the fasting group at 2 a.m. and both fed and fasted mice were euthanized by cervical dislocation at 8–9 p.m. resulting in 18 h of fasting for the fasting group.

For both studies, trunk blood was collected and liver was quickly removed, snap-frozen in liquid nitrogen and stored at − 80 °C until further analyses. Plasma was obtained by centrifugation of the blood at 2600 g and 4 °C for 15 min and stored at − 80 °C. Liver samples were crushed in liquid nitrogen to ensure homogeneity.

### Analyses

#### Plasma substrates

The plasma free fatty acid concentration was measured using the NEFA-HR kit (WAKO Diagnostics GmbH, Germany) according to the manufacturer.

Plasma glucose was determined as glycosyl units measured fluorometrically as previously described [27].

Plasma β-hydroxybutyrate was determined by using the FreeStyle Precision Pro β-ketone monitoring system (Abbott, USA) according to manufacturer guidelines.

#### Hepatic glycogen

Hepatic glycogen content was determined from ~ 10-mg crushed liver samples by boiling the liver samples for 2 h in HCl to hydrolyze glycogen to glycosyl units. NaOH was added for neutralization and hepatic glycogen was determined as glycosyl units measured fluorometrically as previously described [27].

#### Hepatic triglycerides

Hepatic triglyceride content was determined from ~ 20-mg crushed liver tissue as the amount of free glycerol produced after hydrolyzation of triglycerides by ethanolic KOH. Free glycerol was measured using free glycerol reagent (Sigma-Aldrich, Denmark) leading to the production of a quinoneimine dye that was spectrophotometrically detected at 540 nm in a Multiskan (Multiskan FC, Thermo Scientific, USA).

#### Hepatic CS and HAD activity

Maximal hepatic citrate synthase (CS) and hepatic L-3-hydroxy-CoA dehydrogenase (HAD) activity was determined in homogenate obtained by homogenization of liver tissue in phosphate buffer as previously described (Essen-Gustavsson and Henriksson, 1984) using a tissue lyser (TissueLyser II, Qiagen, Germany). CS activity was determined according to manufacturer’s protocol (Sigma-Aldrich, St Louis, MO, USA) by kinetically measuring the absorbance at 405 nm (Multiskan FC, Thermo Scientific, USA) at baseline and after addition of oxaloacetate. HAD activity was determined by kinetically measuring the conversion of NADH to NAD as the change in fluorescence (excitation 355 nm and emission 460 nm) before and after addition of S-Acetoacetyl-CoA. Both CS and HAD activity were normalized to tissue weight.

#### RNA isolation and reverse transcription

Crushed liver tissue (~ 15 mg) was homogenized for 2 min at 30 s^−1^ in a tissue lyser (TissueLyser II, Qiagen, Germany) and total RNA was isolated as previously described [33] by use of a modified guanidium thiocyanate-phenol-chloroform extraction method [7]. RNA concentration and purity of samples were determined spectrophotometrically using a Nanodrop (NanoDrop 1000, Thermo Fisher Scientific). Reverse transcription was performed on 3 μg RNA using Superscript II RNase H^−^ and oligo-dT (Invitrogen, Carlsbad, CA, USA) as previously described [33].

#### Real-time PCR

To determine mRNA content of selected genes, real-time PCR was performed using the ABI-7900 Detection System (Applied Biosystems, Forster City, CA, USA). Primers and 5′-6-carboxyfluorescein (FAM)/3′-6-carboxy – N,N,N′,N′-tetramethylrhodamine (TAMRA) labeled Taqman probes were designed using Primer Express 3.0 software (Applied Biosystems) (Table 1) and were obtained from TAG Copenhagen (Copenhagen, Denmark). Real-time PCR was run in triplicates using Universal Mastermix (Applied Biosystems) with a reaction volume of 10 μl. The cycle threshold for each sample was converted to an arbitrary amount of target mRNA using a standard curve produced from a serial dilution of a pooled sample. Target gene mRNA content was normalized to total single stranded (ss) DNA determined for each sample using Oligreen reagent (Molecular Probes, Leiden, the Netherlands) as previously described [28]. Due to low cDNA content in a few samples of the exercise study, these were omitted from mRNA analyses leading to a lower sample size in some groups.

**Table 1:** Probe and primer sequences used for real-time PCR

Gene	Forward primer	Reverse primer	Probe
HSP72	5’ GATCACGGTGCCAGCCTATT 3’	5’ CGTGGGCTCATTGATTATTCTCA 3’	5’ TCAGCGGCAAGCCACCAAGGAT 3’
PGC-1α	5’ CTCCCTTGTATGTGAGATCACGTT 3’	5’ TGCGGTATTCATCCCTCTTGA 3’	5’ ACAGCCGTAGGCCCAGGTACGACA 3’
sXBP1	5’ TCTGCTGAGTCCGCAGCAGGT 3’	5’ TGCCCAAAAGGATATCAGACTCA 3’	5’ CCCATGGACTCTGACACTGTTGCCTCTT 3’

#### Lysate preparation

Crushed liver tissue (~ 30 mg) was homogenized in ice-cold buffer (10% glycerol, 20 mM Na-pyrophosphate, 150 mM NaCl, 50 mM HEPES, 1% NP-40, 20 mM, β-glycerophosphate, 10 mM NaF, 1 mM EDTA, 1 mM EGTA, 20 μg/ml aprotinin, 10 μg/ml leupeptin, 2 mM Na_3_VO_4_, and 3 mM benzamidine, pH 7.4) as previously described [4] using a tissue lyser (TissueLyser II, QIAGEN, Germany). Samples were rotated end over end for 1 h and lysate was generated by 20-min centrifugation at 16000 *g* and 4 °C. Protein concentration of each sample was determined by the bicinchoninic acid assay (Thermo Fisher Scientific) and samples were prepared with sample buffer containing sodium dodecyl sulfate (SDS) to yield a protein concentration of 2 μg/μl. Samples were boiled for 3 min at 96 °C before protein analyses except for OXPHOS.

#### SDS-PAGE and western blotting

**Fig. 1:**
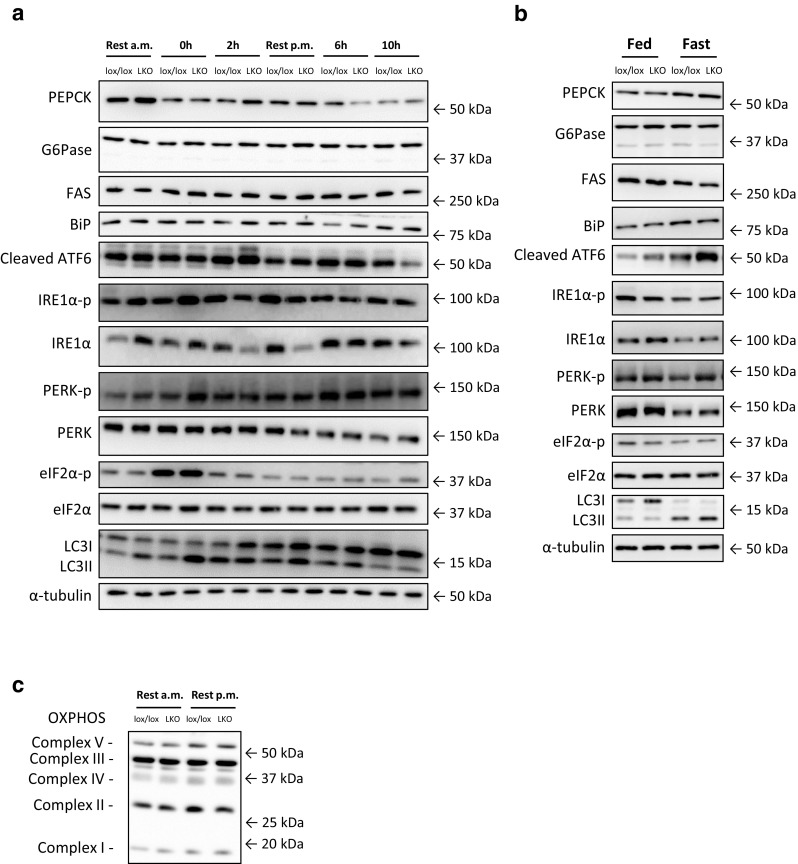
Representative western blots from the exercise (**a**) and fasting (**b**) study. PEPCK, phosphoenolpyruvate carboxykinase; G6Pase, glucose-6-phosphatase; FAS, fatty acid synthase; BiP, binding immunoglobulin protein; ATF6, activating transcription factor 6; IRE1α, inositol-requiring enzyme 1 alpha; PERK, protein kinase-like ER-kinase; eIF2α, eukaryotic translation initiation factor 2 alpha; LC3, microtubule-associated protein 1 light chain 3. In addition, representative western blots of OXPHOS in resting mice (**c**)

Proteins were separated with SDS-PAGE and transferred to a polyvinylidene fluoride membrane (Immobilon-P Transfer Membranes; Millipore) by semi-dry blotting. Membranes were blocked in 3% fish gel for 1 h and incubated overnight at 4 °C with primary antibodies against BiP, PERK, PERK^Thr980^ phosphorylation, eIF2α, eIF2α^Ser51^ phosphorylation, FAS, LC3 (#3177, #3192, #3179, #9722, #9721, #3180, and #4108, respectively, Cell Signaling Technologies, Danvers, MA, USA), IRE1α, IRE1α^Ser724^ phosphorylation, OXPHOS (ab37073, ab48187, and ab110413, respectively, Abcam, Cambridge, UK), PEPCK (10004943; Cayman Chemicals, MI, USA), G6Pase (27198; Santa Cruz Biotechnology, TX, USA), and ATF6 (#IMG-273, IMGENEX, San Diego, CA, USA). After incubation with species-specific horse-radish peroxidase-conjugated secondary antibody (DAKO, Denmark), the protein or phosphorylation of interest was visualized using an ImageQuant LAS 4000 imaging system and quantified with a ImageQuant TL 8.1 software (GE Healthcare, Freiburg, Germany). Protein content and phosphorylation are expressed relative to a pooled sample loaded on each side of the gel. No effect of genotype or intervention was observed for α-tubulin, supporting equal protein content in all samples. Representative blots of all proteins measured are shown in Fig. 1 with samples loaded in the same order as depicted on the graphs.

## Statistics

All data are presented as means ± standard error (SE). A two-way analysis of variance (ANOVA) was used to test the effect of exercise and genotype as well as interaction between exercise and genotype for the early time points (rest a.m., 0 and 2 h) and late time points (rest p.m., 6 h and 10 h) separately. Furthermore, a two-way ANOVA was used to test the effects of time of the day (rest a.m. and rest p.m.) and genotype. Moreover, a one-way ANOVA was applied to test the effect of exercise within each genotype separately. In addition, a two-way ANOVA was used to test the effect of fasting and genotype as well as interaction between fasting and genotype. Data were logarithmically transformed before applying the ANOVA if equal variance test failed. The Student Newman Keuls post-hoc test was used to locate significant differences. In the fasting study, the effect of fasting on PGC-1α mRNA in the lox/lox mice was tested with a *t* test. Throughout, *p* < 0.05 is considered significant. Statistical analyses were performed using SigmaPlot 13.0 (Systat Software, Chicago, I, USA).

## Results

### Oxidative capacity

#### Hepatic CS activity, HAD activity, and OXPHOS protein

To evaluate the impact of PGC-1α on the hepatic oxidative capacity, CS and HAD activity as well as OXPHOS protein was determined at rest. Hepatic CS and HAD activity as well as OXPHOS protein content was similar between genotypes determined in the a.m. resting group (Table 2).

**Table 2:** Activity and protein content of metabolic enzymes. Hepatic citrate synthase (CS) activity, L-3-hydroxy-CoA dehydrogenase (HAD) activity, and OXPHOS protein content in liver-specific PGC-1α knockout (LKO) and littermate floxed (lox/lox) mice at rest. Values are presented as means ± standard error (SE); n = 9–10

	lox/lox	LKO
CS activity (μmol min^−1^ g wet weight^−1^)	25.2 ± 1.0	23.2 ± 0.6
HAD activity (μmol min^−1^ g wet weight^−1^)	179.6 ± 5.5	177.5 ± 5.6
OXPHOS protein (AU)	1.2 ± 0.02	1.2 ± 0.03

### Exercise study

#### Food intake

Food intake was not different between rest and 2 h in lox/lox mice (0.12 ± 0.07 and 0.10 ± 0.03, rest and 2 h, respectively), but was ≈ 12-fold higher (*p* < 0.05) 2 h into recovery than at rest in LKO mice (0.03 ± 0.03 and 0.25 ± 0.07, rest and 2 h, respectively). In addition, food intake was ≈ 2-fold higher (*p* < 0.05) in LKO than in lox/lox mice at 2 h. Food intake was ≈ 1.6-fold higher (*p* < 0.05) 10 h into recovery than at rest in both genotypes (lox/lox: 0.60 ± 0.11 and 0.99 ± 0.19, rest and 10 h, respectively. LKO: 0.58 ± 0.06 and 0.92 ± 0.12, rest and 10 h, respectively) and there was no difference between genotypes at the late recovery time points. Food intake was ≈ 5 and ≈ 20-fold higher (*p* < 0.05) in rest p.m. than rest a.m. in both genotypes (data not shown).

#### Plasma substrates

The plasma glucose concentration was unchanged in response to exercise in both genotypes, but there was an overall genotype difference (*p* < 0.05) in the rest, 6 and 10 h groups (Table 3).

**Table 3:** Plasma glucose and free fatty acid (FFA) concentration in liver-specific PGC-1α knockout (LKO) and littermate floxed (lox/lox) mice at rest and 0, 2, 6, and 10 h into recovery from 1-h treadmill running. Resting mice were either euthanized at time points corresponding to 0 and 2h (Rest a.m.) or 6 and 10 h (Rest p.m.). Values are presented as means ± standard error (SE); n = 9–10. A single asterisk indicates a significant difference from Rest within given genotype, p < 0.05. A single dagger indicates a significant difference from Rest a.m. within given genotype, p < 0.05

	Rest a.m.		0 h		2 h		Rest p.m.		6 h		10 h	
	lox/lox	LKO	lox/lox	LKO	lox/lox	LKO	lox/lox	LKO	lox/lox	LKO	lox/lox	LKO
Glucose (mmol L^−1^)	9.3 ± 0.3	9.2 ± 0.3	10.1 ± 0.4	9.4 ± 0.3	9.1 ± 0.3	9.2 ± 0.3	8.7 ± 0.5	9.0 ± 0.3	8.5 ± 0.5	9.4 ± 0.3	7.8 ± 0.2	8.7 ± 0.3
Free fatty acids (mmol L^−1^)	0.7 ± 0.03	0.7 ± 0.1	0.7 ± 0.04	0.7 ± 0.03	0.7 ± 0.1	0.6 ± 0.1	0.5 ± 0.1†	0.5 ± 0.05†	0.5 ± 0.1	0.6 ± 0.1	0.4 ± 0.1	0.3 ± 0.04*
β-hydroxybutyrate (mmol L^−1^)	0.1 ± 0.02	0.2 ± 0.04	0.7 ± 0.2*	0.8 ± 0.1*	0.3 ± 0.1	0.2 ± 0.1	0.1 ± 0.02	0.1 ± 0.04	0.1 ± 0.02	0.1 ± 0.02	0.1 ± 0.02	0.1 ± 0.02

The plasma free fatty acid concentration was unchanged after exercise in lox/lox mice, but ≈ 40% lower (*p* < 0.05) at 10 h than rest in LKO mice. In both genotypes, the plasma free fatty acid concentration was ≈ 30% lower (*p* < 0.05) in rest p.m. than rest a.m. There was no genotype difference in the plasma free fatty acid concentration in any of the groups (Table 3).

The plasma β-hydroxybutyrate concentration was 5–7.5-fold higher (*p* < 0.05) immediately after exercise than at rest in both lox/lox and LKO mice, and there was no difference between genotypes in any of the groups (Table 3).

#### Glycogen and triglycerides

**Fig. 2:**
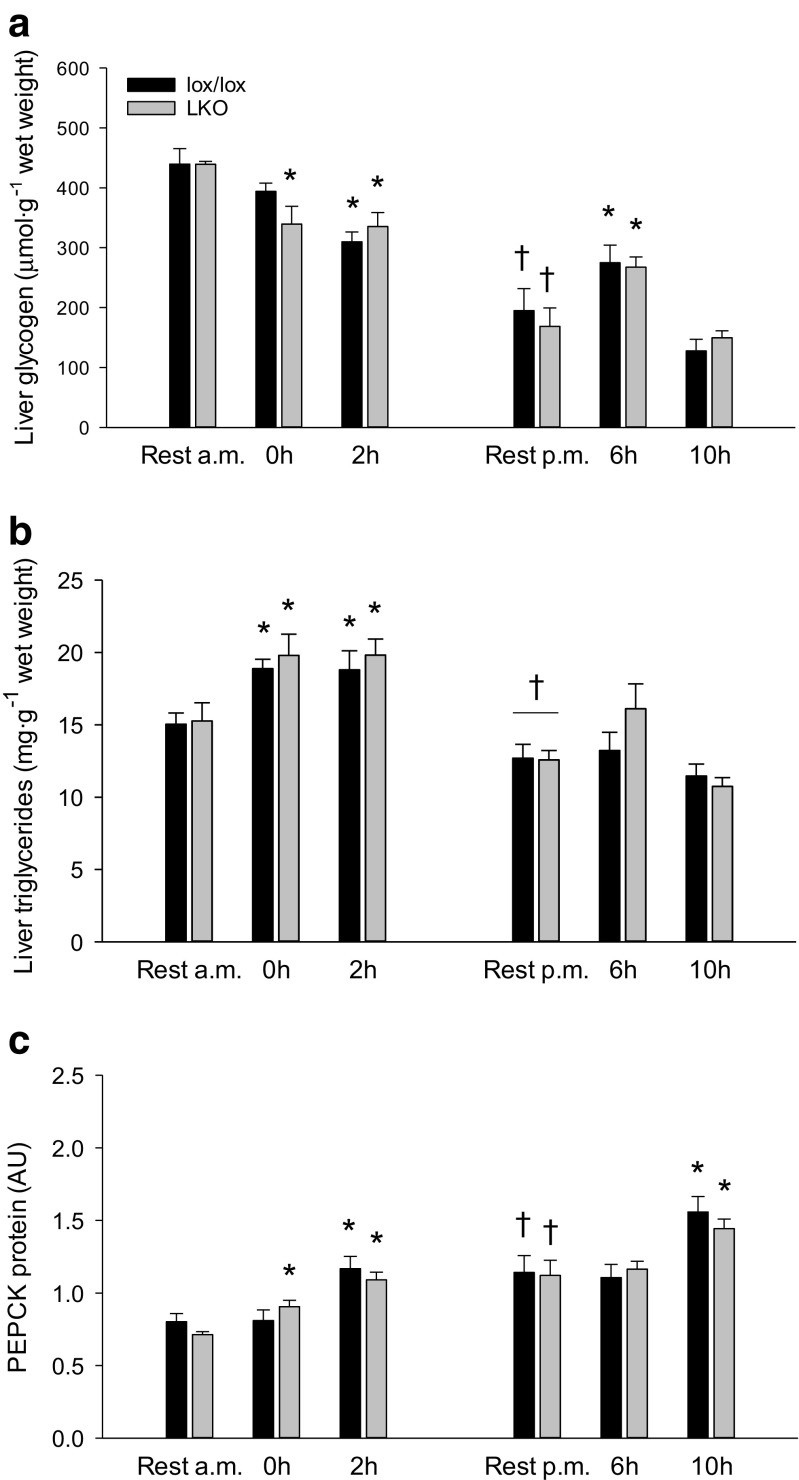
Hepatic glycogen (**a**), triglyceride (**b**), and PEPCK protein (**c**) content in liver-specific PGC-1α knockout (LKO) and littermate floxed (lox/lox) mice at rest and 0, 2, 6, and 10 h into recovery from 1-h treadmill running. Resting mice were either euthanized at time points corresponding to 0 and 2 h (rest a.m.) or 6 and 10 h (rest p.m.). Protein is given in arbitrary units (AU). Values are presented as means ± standard error (SE); *n* = 8–10. A single asterisk is significantly different from rest within given genotype, *p* < 0.05. A single dagger is significantly different from rest a.m. within given genotype, *p* < 0.05. Horizontal line indicates a main effect, *p* < 0.05

Hepatic glycogen content in lox/lox mice was ≈ 30% lower (*p* < 0.05) at 2 h than rest and ≈ 1.4-fold higher (*p* < 0.05) at 6 h than rest as well as ≈ 50% lower (*p* < 0.05) at rest p.m. than rest a.m. In LKO mice, glycogen content in the liver was ≈ 25% lower (*p* < 0.05) immediately after exercise and at 2 h than rest. There was no difference in hepatic glycogen content between genotypes in any of the groups (Fig. 2a).

The hepatic triglyceride content was ≈ 1.4-fold higher (*p* < 0.05) immediately after exercise than in the rest group and ≈ 20% lower (*p* < 0.05) at rest p.m. than rest a.m. in both genotypes. There was no difference in hepatic triglyceride content between genotypes in any of the groups (Fig. 2b).

#### PEPCK, G6Pase, and FAS protein

Hepatic PEPCK protein content was in both genotypes ≈ 1.5-fold higher (*p* < 0.05) at 2 and 10 h than the corresponding rest group as well as ≈ 1.4-fold higher (*p* < 0.05) at rest p.m. than rest a.m. Moreover, PEPCK protein in LKO mice was ≈ 1.2-fold higher (*p* < 0.05) immediately after exercise than at rest. In addition, there was no difference in hepatic PEPCK protein content between genotypes in any of the groups (Fig. 2c).

There was no difference in hepatic G6Pase protein content between groups in any of the genotypes, and no genotype difference was evident (Table 4).

**Table 4:** Hepatic G6Pase and FAS protein content in liver-specific PGC-1α knockout (LKO) and littermate floxed (lox/lox) mice at rest and 0, 2, 6, and 10 h into recovery from 1-h treadmill running and OXPHOS protein content from resting lox/lox and LKO mice. Resting mice were either euthanized at time points corresponding to 0 and 2 h (Rest a.m.) or 6 and 10 h (Rest p.m.). Protein content is given in arbitrary units (AU). Values are presented as means ± standard error (SE); n = 8–10

	Rest a.m.		0 h		2 h		Rest p.m.		6 h		10 h	
	lox/lox	LKO	lox/lox	LKO	lox/lox	LKO	lox/lox	LKO	lox/lox	LKO	lox/lox	LKO
G6Pase protein (AU)	1.0 ± 0.1	1.0 ± 0.1	0.9 ± 0.1	0.9 ± 0.1	1.1 ± 0.1	1.0 ± 0.1	0.9 ± 0.1	0.9 ± 0.1	0.9 ± 0.1	0.9 ± 0.1	0.9 ± 0.1	1.0 ± 0.05
FAS protein (AU)	1.4 ± 0.1	1.6 ± 0.1	1.4 ± 0.1	1.4 ± 0.1	1.4 ± 0.1	1.3 ± 0.1	1.4 ± 0.1	1.4 ± 0.1	1.4 ± 0.1	1.5 ± 0.1	1.4 ± 0.1	1.3 ± 0.1

Hepatic FAS protein content was not different between groups in either of the genotypes and not different between genotypes in any of the groups (Table 4).

#### UPR protein and phosphorylation

**Fig. 3:**
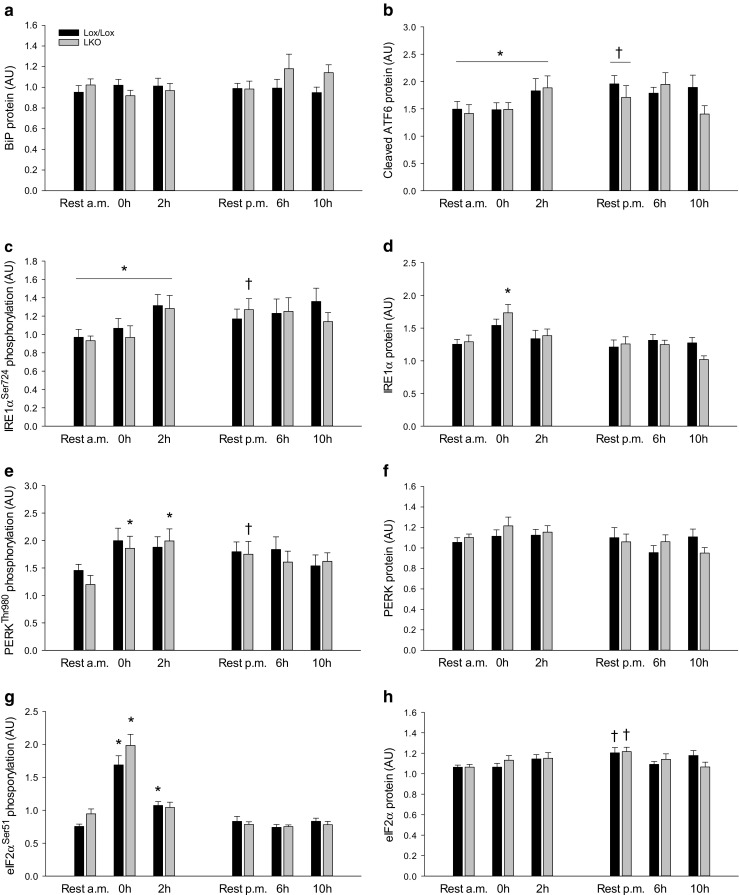
Hepatic BiP protein (**a**), cleaved ATF6 protein (**b**), IRE1α^Ser924^ phosphorylation (**c**), IRE1α protein (**d**), PERK^Thr980^ phosphorylation (**e**), PERK protein (**f**), eIF2α^Ser51^ phosphorylation (**g**), and eIF2α protein (**h**) content in liver-specific PGC-1α knockout (LKO) and littermate floxed (lox/lox) mice at rest and 0, 2, 6, and 10 h into recovery from 1-h treadmill running. Resting mice were either euthanized at time points corresponding to 0 and 2 h (rest a.m.) or 6 and 10 h (rest p.m.). Protein content and phosphorylation are given in arbitrary units (AU). Values are presented as means ± standard error (SE); *n* = 8–10. A single asterisk is significantly different from rest within given genotype, *p* < 0.05. A single dagger is significantly different from rest a.m. within given genotype, *p* < 0.05. Horizontal line indicates a main effect, *p* < 0.05

Hepatic BiP protein was not different between groups in either genotype and there was no genotype difference in any of the groups (Fig. 3a).

There was an overall difference (*p* < 0.05) in hepatic cleaved ATF6 protein content between rest, 0 and 2 h as well as between rest p.m. and rest a.m.. There was no difference in cleaved ATF6 in the liver between genotypes in any of the groups (Fig. 3b).

There was an overall difference (*p* < 0.05) in hepatic IRE1α phosphorylation between rest, 0 and 2 h. Moreover, hepatic IRE1α phosphorylation was ≈ 1.3-fold higher (*p* < 0.05) in rest p.m. than rest a.m. in LKO mice, while there was no difference in IRE1α phosphorylation between genotypes in any of the groups (Fig. 3c). IRE1α protein content in the liver was not different between groups in lox/lox mice, but was ≈ 1.3-fold higher (*p* < 0.05) immediately after exercise than rest in LKO mice. There was no difference in IRE1α protein between genotypes in any of the groups (Fig. 3d).

Hepatic PERK phosphorylation in lox/lox mice was not different between groups, but was ≈ 1.6-fold higher (*p* < 0.05) 0 and 2 h after exercise than at rest in LKO mice. In addition, PERK phosphorylation in the liver was ≈ 1.4-fold higher (*p* < 0.05) in rest p.m. than rest a.m. within LKO. There was no difference in PERK phosphorylation between genotypes in any of the groups (Fig. 3e). PERK protein content in the liver was not different between groups in either genotype, and there was no genotype difference in PERK protein content in any of the groups (Fig. 3f).

Hepatic eIF2α phosphorylation was ≈ 2.2-fold higher (*p* < 0.05) immediately after exercise than rest in both genotypes, and there was no difference between genotypes in any of the groups (Fig. 3g). Protein content of eIF2α was not different between resting and exercising groups in either genotype, but was ≈ 1.1-fold higher (*p* < 0.05) in rest p.m. than rest a.m. in both genotypes. There was no difference in eIF2α protein between genotypes in any of the groups (Fig. 3h).

#### Downstream UPR mRNA

**Fig. 4:**
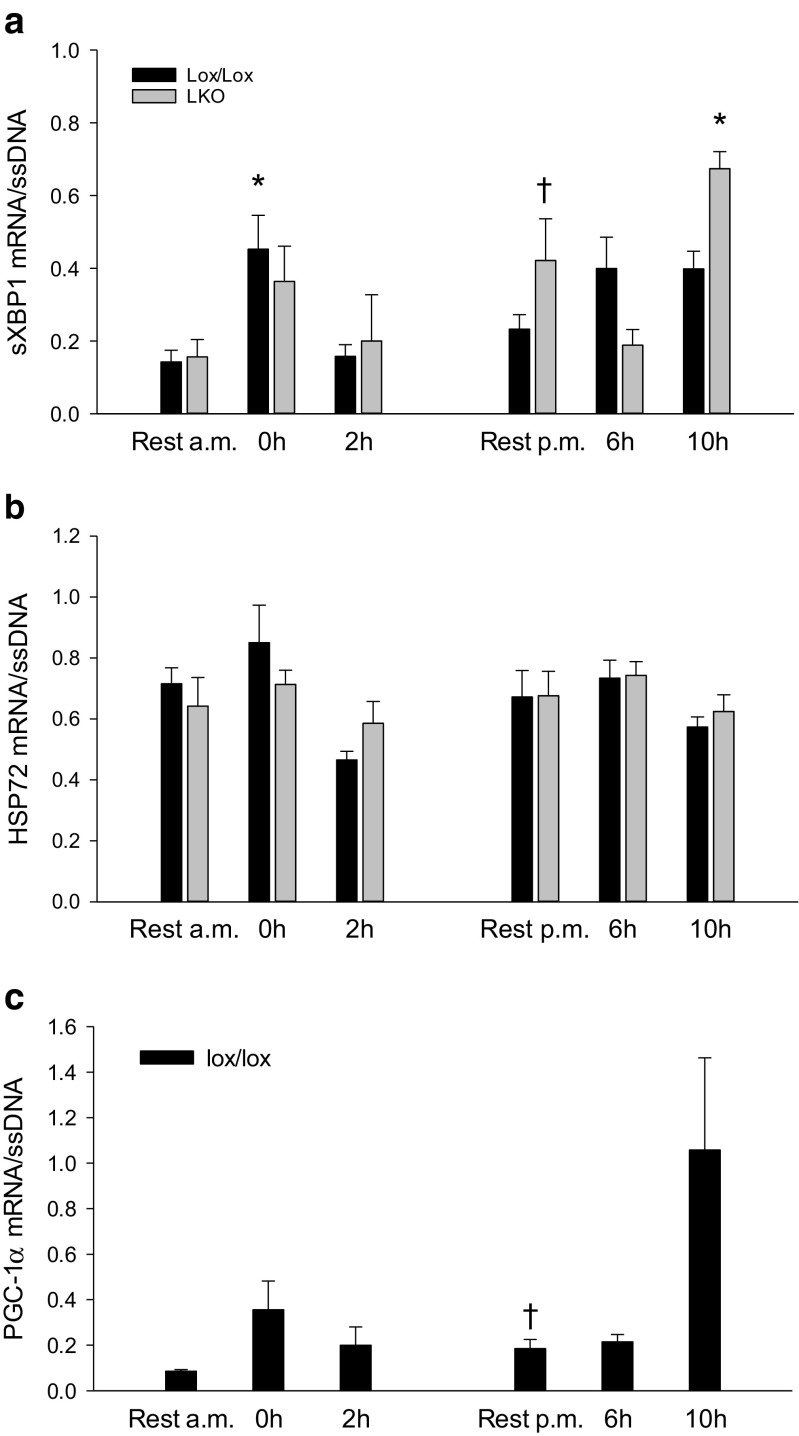
Hepatic sXBP1 (**a**) and HSP72 (**b**) mRNA content in liver-specific PGC-1α knockout (LKO) and littermate floxed (lox/lox) mice and PGC-1α mRNA in lox/lox mice (**c**) at rest and 0, 2, 6, and 10 h into recovery from 1-h treadmill running. Resting mice were either euthanized at time points corresponding to 0 and 2 h (rest a.m.) or 6 and 10 h (rest p.m.). The target mRNA is normalized to single stranded (ss) DNA. Values are presented as means ± standard error (SE); *n* = 6–9. A single asterisk is significantly different from rest within given genotype, *p* < 0.05. A single dagger is significantly different from rest a.m. within given genotype, *p* < 0.05

The mRNA content of sXBP1 was in lox/lox mice ≈ 3-fold higher (*p* < 0.05) immediately after exercise than rest. In LKO mice, the sXBP1 mRNA content was ≈ 1.5-fold higher (*p* < 0.05) at 10 h of recovery than rest and ≈ 2.5-fold higher (*p* < 0.05) in rest p.m. than rest a.m. There was no genotype difference in sXBP1 mRNA in any of the groups (Fig. 4a).

Hepatic HSP72 mRNA content was not different between groups in either genotype, and there was no difference in HSP72 mRNA content between genotypes in any of the groups (Fig. 4b).

#### PGC-1α mRNA

Hepatic PGC-1α mRNA content in lox/lox mice was not different between groups, but was 2-fold higher (*p* < 0.05) in rest p.m. than rest a.m. (Fig. 4c).

#### Autophagy

**Fig. 5:**
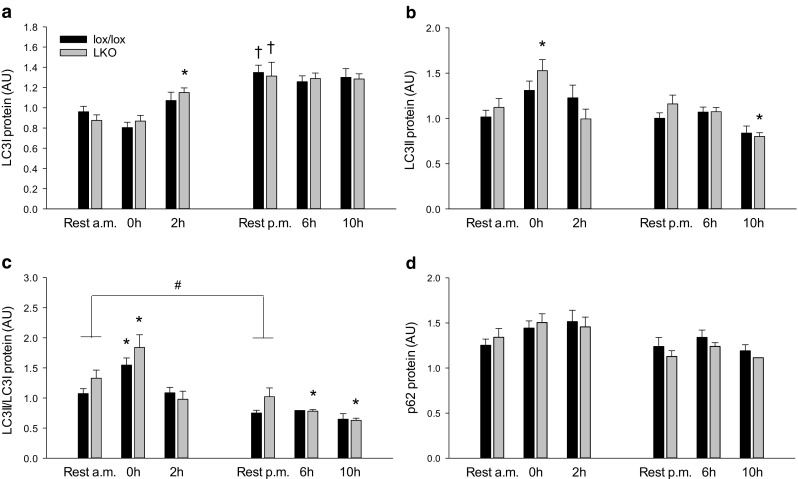
Hepatic LC3I (**a**), LC3II (**b**), LC3II/LC3I protein ratio (**c**) and p62 protein (**d**) in liver-specific PGC-1α knockout (LKO) and littermate floxed (lox/lox) mice at rest and 0, 2, 6, and 10 h into recovery from 1-h treadmill running. Resting mice were either euthanized at time points corresponding to 0 and 2 h (rest a.m.) or 6 and 10 h (rest p.m.). Protein content is given in arbitrary units (AU). Values are presented as means ± standard error (SE); *n* = 8–10. A single asterisk is significantly different from rest within given genotype, *p* < 0.05. A single dagger is significantly different from rest a.m. within given genotype, *p* < 0.05. A single number sign is significantly different from lox/lox within given group, *p* < 0.05. Horizontal line indicates a main effect, *p* < 0.05

Hepatic LC3I protein content was not different between resting and exercising groups in lox/lox mice, but 1.3-fold higher (*p* < 0.05) 2 h into recovery than at rest in LKO mice. In both genotypes, LC3I protein was ≈ 1.6-fold higher (*p* < 0.05) at rest p.m. than rest a.m. in both genotypes. There was no difference in LC3I protein content between genotypes in any of the groups (Fig. 5a).

Protein content of LC3II in the liver was not different between groups in lox/lox mice, but was in LKO mice ≈ 1.3-fold higher (*p* < 0.05) immediately after exercise than rest. There was no difference in LC3II protein content between genotypes in any of the groups (Fig. 5b).

The LC3II/LC3I protein ratio in the liver was ≈ 1.4-fold higher (*p* < 0.05) immediately after exercise than rest in both genotypes. Moreover, the hepatic LC3II/LC3I protein ratio was ≈ 25% lower (*p* < 0.05) 6 and 10 h after exercise than rest in LKO. In addition, there was an overall genotype difference (*p* < 0.05) in the rest groups (Fig. 5c).

Hepatic p62 protein content was not different between groups in either genotype, and there was no difference in p62 protein content between genotypes in any of the groups (Fig. 5d).

### Fasting study

#### Plasma substrates

The plasma glucose concentration was 35–40% lower (*p* < 0.05) in fasted mice than in fed mice in both genotypes, and there was no difference between genotypes (Table 5).

**Table 5:** Plasma glucose, free fatty acid (FFA) and β-hydroxybutyrate concentration in fed (Fed) and 18-h fasted (Fast) liver-specific PGC-1α knockout (LKO) and littermate floxed (lox/lox) mice. Values are presented as means ± standard error (SE); n = 7–8. A single asterisk indicates a significant difference from Fed within given genotype, p < 0.05

	Fed		Fast	
	lox/lox	LKO	lox/lox	LKO
Glucose (mmol L^−1^)	9.3 ± 0.6	9.3 ± 0.4	5.9 ± 0.3*	5.6 ± 0.1*
Free fatty acids (mmol L^−1^)	0.3 ± 0.1	0.1 ± 0.03	1.3 ± 0.1*	1.4 ± 0.1*
β-hydroxybutyrate (mmol L^−1^)	0.2 ± 0.03	0.2 ± 0.05	2.7 ± 0.1*	2.2 ± 0.2*

In both genotypes, the plasma free fatty acid (FFA) concentration was 5–9-fold higher (*p* < 0.05) in the fasted state than in the fed state, and there was no difference between genotypes (Table 5).

Fasting resulted in 13–19-fold higher (*p* < 0.05) plasma β-hydroxybutyrate concentration than in fed mice in both genotypes, and there was no difference between genotypes (Table 5).

#### Glycogen and triglycerides

**Fig. 6:**
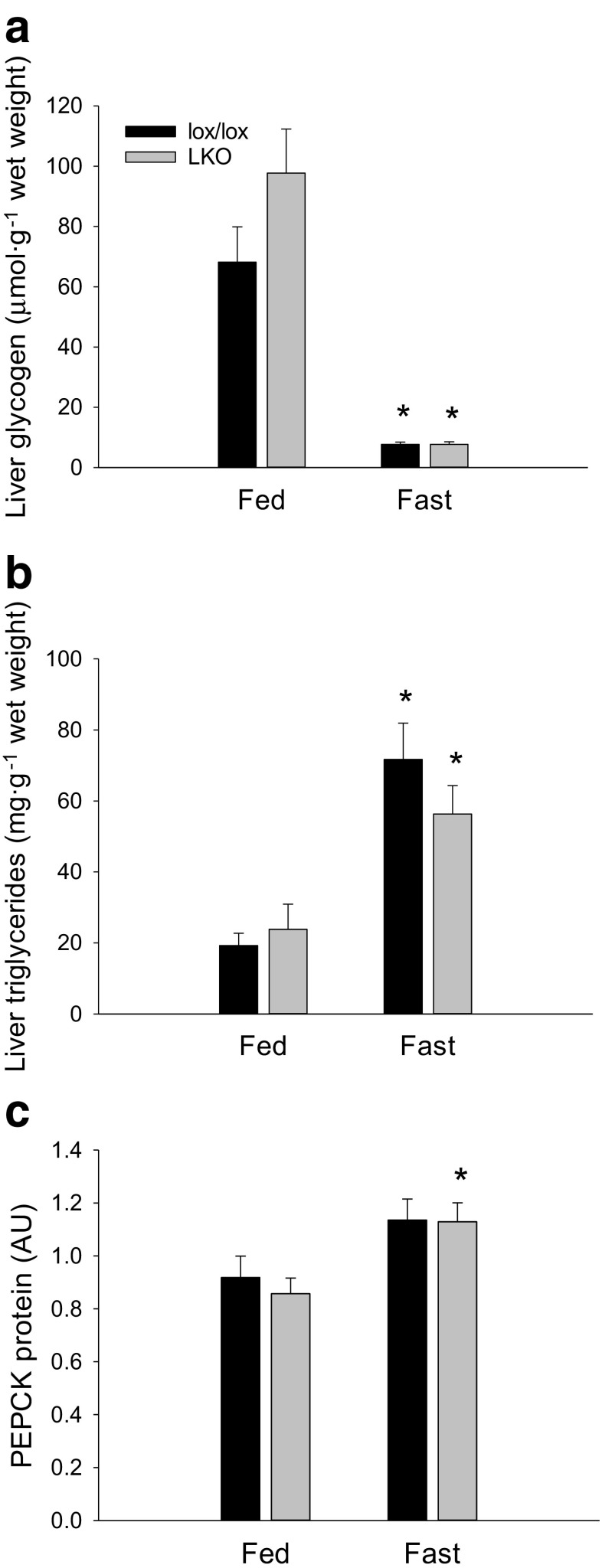
Hepatic glycogen (**a**), triglyceride (**b**), and PEPCK protein (**c**) content in fed (Fed) and 18 h fasted (Fast) liver-specific PGC-1α knockout (LKO), and littermate floxed (lox/lox) mice. Protein is given in arbitrary units (AU). Values are presented as means ± standard error (SE); *n* = 7–8. A single asterisk is significantly different from Fed within given genotype, *p* < 0.05

Liver glycogen was 90–95% lower (*p* < 0.05) in fasted mice than in fed mice in both genotypes and there was no difference between genotypes (Fig. 6a).

Triglyceride content in the liver was 2–3.5-fold higher (*p* < 0.05) in the fasted state than in the fed state in both genotypes, and there was no difference between genotypes (Fig. 6b).

#### PEPCK, G6Pase, and FAS protein

Hepatic PEPCK protein content was not different between fed and fasted lox/lox mice, but was ≈ 1.2-fold higher (*p* < 0.05) in the fasted state than the fed state in LKO mice. There was no genotype difference in any of the groups (Fig. 6c).

Hepatic G6Pase protein and FAS protein were not different between fed and fasted mice in either genotype, and there was no genotype difference in any of the groups (Table 6).

**Table 6:** Hepatic G6Pase and FAS protein content in fed (Fed) and 18-h fasted (Fast) liver-specific PGC-1α knockout (LKO) and littermate floxed (lox/lox) mice. Protein content is given in arbitrary units (AU). Values are presented as means ± standard error (SE), n = 8

	Fed		Fast	
	lox/lox	LKO	lox/lox	LKO
G6Pase protein (AU)	1.0 ± 0.05	1.1 ± 0.1	1.2 ± 0.1	1.1 ± 0.1
FAS protein (AU)	1.1 ± 0.1	1.2 ± 0.2	1.0 ± 0.1	0.9 ± 0.1

#### UPR protein and phosphorylation

**Fig. 7:**
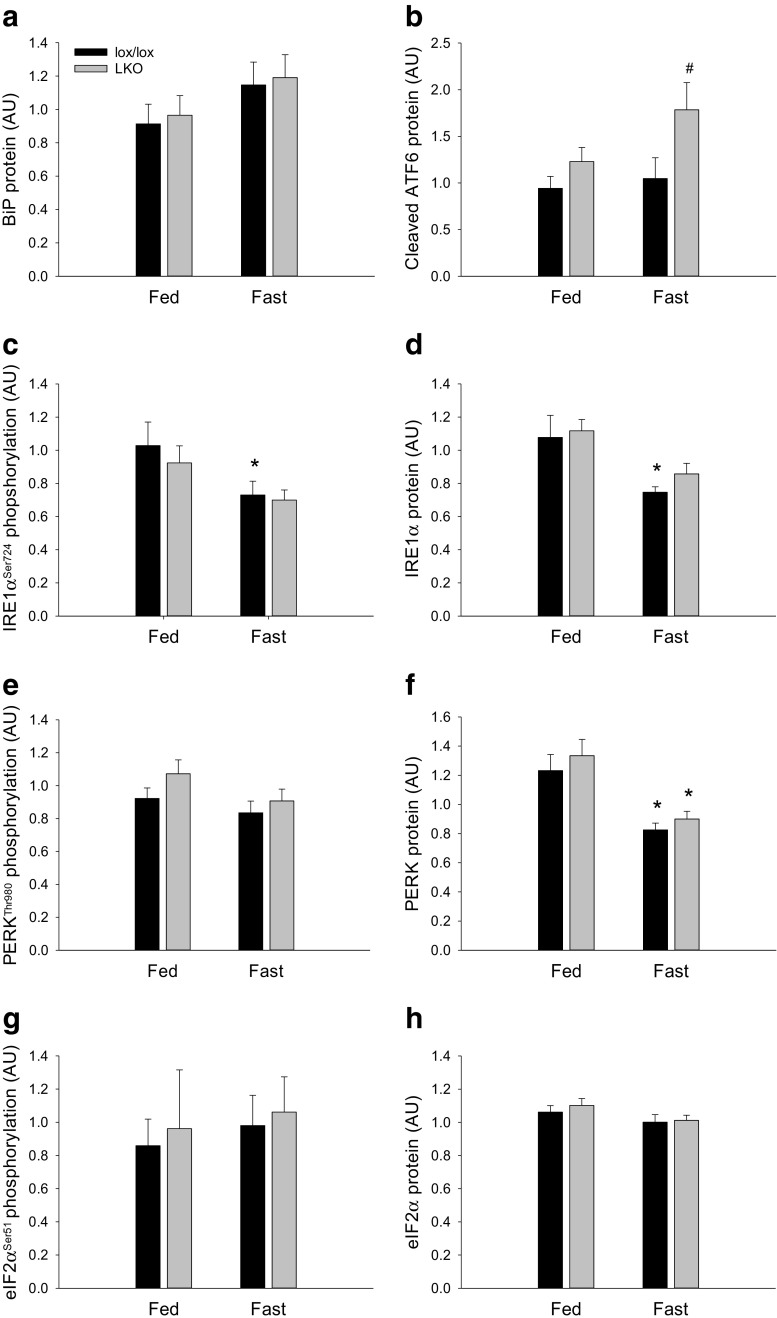
Hepatic BiP protein (**a**), cleaved ATF6 protein (**b**), IRE1α^Ser724^ phosphorylation (**c**), IRE1α protein (**d**), PERK^Thr980^ phosphorylation (**e**), PERK protein (**f**), eIF2α^Ser51^ phosphorylation (**g**), and eIF2α protein (**h**) content in fed (Fed) and 18 h fasted (Fast) liver-specific PGC-1α knockout (LKO) and littermate floxed (lox/lox) mice. Protein is given in arbitrary units (AU). Values are presented as means ± standard error (SE); *n* = 7–8. A single asterisk is significantly different from Fed within given genotype, *p* < 0.05. A single number sign is significantly different from lox/lox within given group, *p* < 0.05

Hepatic BiP protein content was not different between groups in either genotype, and there was no difference between genotypes (Fig. 7a).

Cleaved ATF6 protein content in the liver was not different between groups in either genotype, but was ≈ 1.7-fold higher (*p* < 0.05) in LKO than lox/lox within the fasted groups (Fig. 7b).

Hepatic IRE1α phosphorylation and IRE1α protein content were ≈ 30% lower (*p* < 0.05) in the fasted state than in the fed state in lox/lox mice, whereas there was no difference between fed and fasted LKO mice. There was no genotype difference in either IRE1α phosphorylation or protein content in the liver in any of the groups (Fig. 7c, d).

PERK phosphorylation in the liver was not different between groups in either genotype, but hepatic PERK protein content was ≈ 35% lower (*p* < 0.05) in fasted than fed mice in both genotypes. There was no genotype difference in PERK phosphorylation or protein content in any of the groups (Fig. 7e, f).

Neither hepatic eIF2α phosphorylation nor protein content, the downstream target of PERK, was different between fed and fasted mice of either genotype, and there was no genotype difference in either phosphorylation or protein content of eIF2α in any of the groups (Fig. 7g, h).

#### Downstream UPR mRNA

**Fig. 8:**
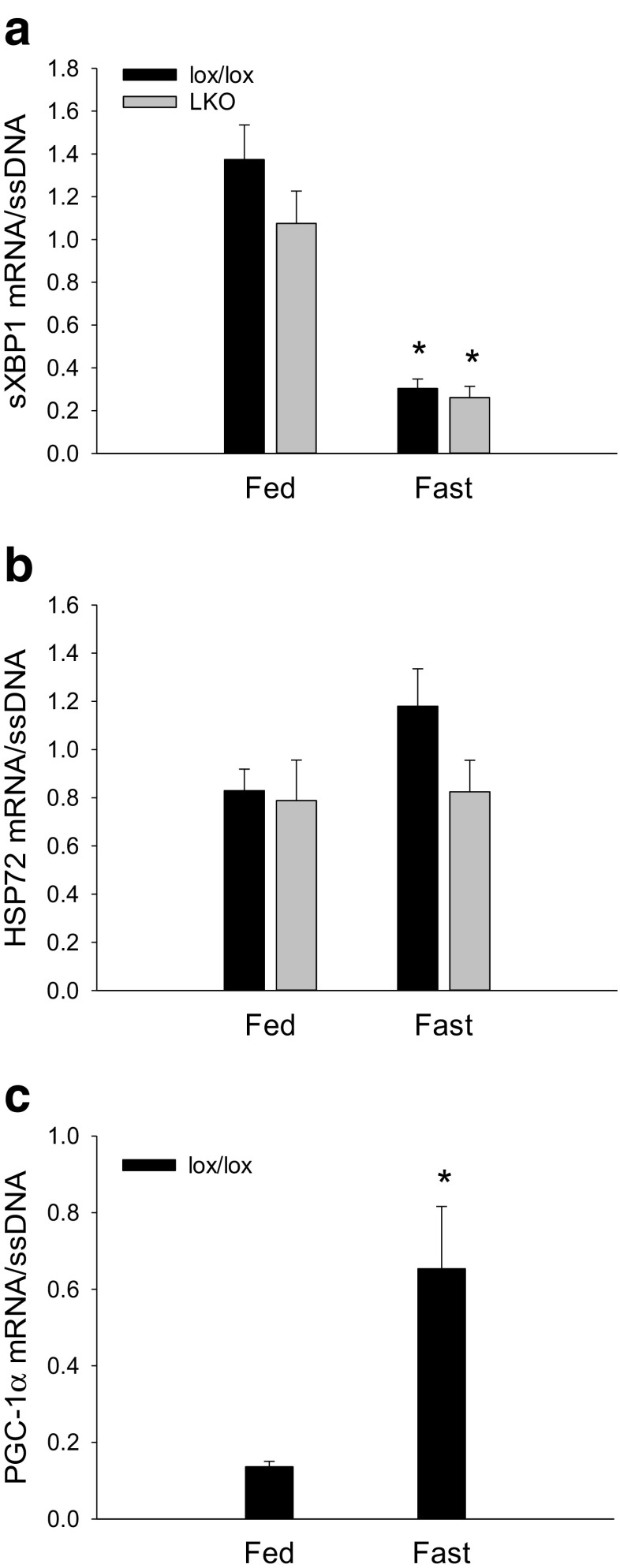
Hepatic sXBP1 (**a**) and HSP72 (**b**) mRNA content in fed (Fed) and 18 h fasted (Fast) liver-specific PGC-1α knockout (LKO) and littermate floxed (lox/lox) mice and PGC-1α mRNA in lox/lox mice (**c**). The target mRNA is normalized to single stranded (ss) DNA. Values are presented as means ± standard error (SE); *n* = 6–8. A single asterisk is significantly different from Fed within given genotype, *p* < 0.05

Hepatic sXBP1 mRNA content was ≈ 80% lower (*p* < 0.05) in the fasted state than in the fed state within both genotypes, and there was no difference in sXBP1 mRNA between genotypes (Fig. 8a).

There was no difference in hepatic HSP72 mRNA content between fed and fasted mice in either genotype, and there was no genotype difference (Fig. 8b).

#### PGC-1α mRNA

Hepatic PGC-1α mRNA content was ≈ 4.5-fold higher (*p* < 0.05) in the fasted state than in the fed state in lox/lox mice (Fig. 8c).

#### Autophagy

**Fig. 9:**
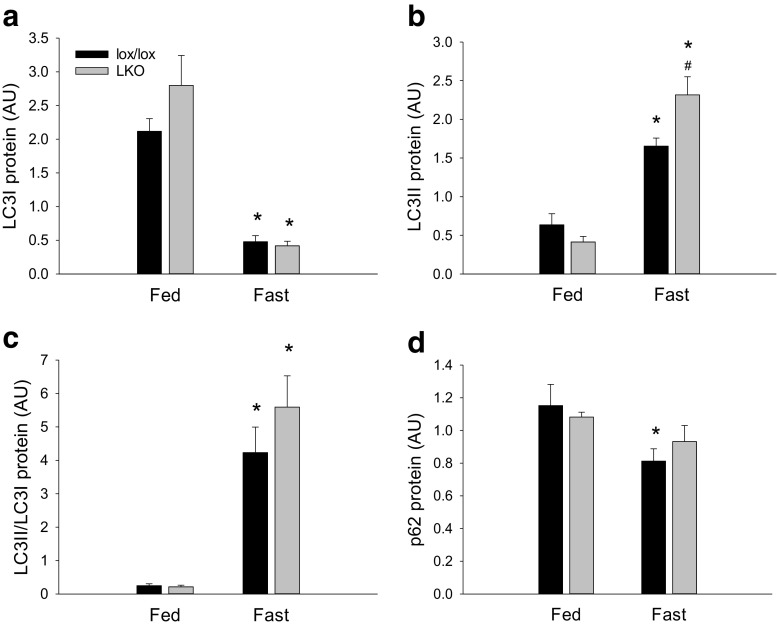
Hepatic LC3I protein (**a**), LC3II protein (**b**), LC3II/LC3I (**c**) and p62 protein (**d**) protein ratio in fed (Fed) and 18 h fasted (Fast) liver-specific PGC-1α knockout (LKO) and littermate floxed (lox/lox) mice. Protein content is given in arbitrary units (AU). Values are presented as means ± standard error (SE); *n* = 7–8. A single asterisk is significantly different from Fed within given genotype, *p* < 0.05. A single number sign significantly different from lox/lox within given group, *p* < 0.05

Hepatic LC3I protein content was 80–85% lower (*p* < 0.05) in fasted mice than in fed mice in both genotypes, and there was no difference in LC3I protein content between genotypes (Fig. 9a).

There was an interaction (*p* < 0.05) between groups and genotype in LC3II protein content. Hepatic LC3II protein content was ≈ 2.6-fold and ≈ 5-fold higher (*p* < 0.05) in fasted than fed mice in lox/lox and LKO mice, respectively. Moreover, LC3II protein content was ≈ 1.4-fold higher (*p* < 0.05) in LKO mice than lox/lox mice in the fasted state (Fig. 9b).

The hepatic LC3II/LC3I protein ratio was 15–19-fold higher (*p* < 0.05) in fasted than in fed mice within both genotypes, and there was no genotype difference in the LC3II/LC3I ratio in any of the groups (Fig. 9c).

Hepatic p62 protein content was ≈ 30% lower (*p* < 0.05) in fasted than fed lox/lox mice only. There was no genotype difference in p62 protein content in any of the groups (Fig. 9d).

## Discussion

The main findings of the present study are that (1) an acute bout of exercise led to upregulation of the hepatic UPR with apparent activation of all three UPR pathways independent of PGC-1α and (2) 18 h of fasting decreased the capacity of the PERK and IRE1α pathway as well as regulated autophagy in the liver of mice independent of PGC-1α. However, hepatic PGC-1α may contribute in regulating basal hepatic autophagy.

The observation that eIF2α phosphorylation increased immediately after exercise is in accordance with our previous study reporting similar increased phosphorylation of PERK and eIF2α in mouse liver immediately after exercise (unpublished, Kristensen et al.). On the other hand, the observed increase in cleaved ATF6 and IRE1α phosphorylation 2 h into recovery from exercise in the present study is a new finding and in contrast to the lack of change in hepatic cleaved ATF6 and IRE1α phosphorylation in the previous study (unpublished, Kristensen et al.). This may be related to the differences in exercise-induced changes in hepatic triglyceride and glycogen content in the present and the previous study (unpublished, Kristensen et al.).

The present study suggests that there was a time-specific activation of the three UPR pathways in the liver with activation of the PERK pathway immediately after exercise and with later activation of the ATF6 and IRE1α pathways. In line with the increased IRE1α phosphorylation is the finding that hepatic sXBP1 mRNA also was elevated after exercise, although the increase in sXBP1 mRNA preceded the increase in IRE1α phosphorylation. Whether undetectable increases in IRE1α phosphorylation or other posttranslational modifications of IRE1α may have induced splicing of XBP1 remains unknown. The increase in sXBP1 mRNA observed in the present study is in contrast to a previously observed transient decrease in sXBP1 mRNA content 2 h into recovery from an exercise bout in mice (unpublished, Kristensen et al.). The discrepancy in the exercise-induced regulation of hepatic UPR between the present and the previous study (unpublished, Kristensen et al.) may be due to different mouse strains, because control mice in the previous study were wild-type mice, whereas the control mice in the present study were PGC-1α floxed. Alternatively, the difference may be related to the slightly higher intensity in the current study than the previous. The lack of increase in HSP72 mRNA in the liver of exercised mice in the present study is in contrast to previous studies reporting a transient increase in mRNA of multiple HSPs including HSP72 in rodent liver in response to exercise [12] (unpublished, Kristensen et al.). This suggests that the exercise-induced stress response was limited to the classical UPR components, which may be due to the liver likely being less challenged during the exercise bout in the present study than the previous acute exercise studies, based on the lack of change in hepatic glycogen and less increased hepatic triglyceride content.

The observation that the protein content of PERK and IRE1α as well as the phosphorylation of IRE1α decreased with 18 h of fasting is in accordance with our previous study reporting downregulation of these UPR pathways with 24 h of fasting (unpublished, Kristensen et al.) and suggests that the activity of these specific UPR pathways are downregulated during fasting. This is supported by the finding that sXBP1 mRNA content was also decreased in the fasted mice in the current study. On the other hand, previous studies have reported activation of the IRE1α and ATF6 pathway in mouse liver with 18–24 h of fasting [6, 36]. Differences in mouse strains and time point of sampling may explain this discrepancy and the present results seem to indicate that the hepatic fasting response can include downregulation of UPR in mice. Taken together, the present study suggests that although exercise and fasting are two states characterized by similar low nutrient availability, they result in differential regulation of the hepatic UPR potentially explained by the different durations of the challenges. Exercise is a relatively short challenge and may therefore induce a transient UPR in order to reestablish ER homeostasis, whereas fasting may spare energy by downregulating processes not necessary for the liver to overcome the nutrient deprivation.

The observed increase in the LC3II/LC3I protein ratio in the liver immediately after exercise is in accordance with previous studies reporting increased LC3II/LC3I ratio in mouse liver post exercise [17] (unpublished, Kristensen et al.) and may indicate enhanced autophagy. On the other hand, this is not supported by unchanged LC3II and p62 protein content with exercise. Moreover, the present observation that LC3I protein content increased 2 h into recovery may suggest that exercise transiently increased the capacity for autophagy. It may be speculated that hepatic autophagy is elevated to provide recycling of cellular components or potentially remove damaged proteins after or during exercise. Insulin has previously been shown to inhibit hepatic autophagy [26], and it could be speculated that the increase in autophagy markers in the resting and recovery groups was a result of food intake and hence elevated plasma insulin levels. However, data from our group has demonstrated that insulin does not increase until 10 h into recovery after an exercise bout [22]. This is in accordance with the present observation that food intake was markedly increased 10 h into recovery. Therefore, it may be speculated that because mice in the rest p.m. group had a higher food intake than the rest a.m. group but similar response in LC3 and p62 protein, food intake (and insulin levels) may not be the major factor regulating autophagy markers in the present study.

The present observation that LC3II and the LC3II/LC3I ratio increased with 18 h of fasting is in accordance with previous studies reporting similar findings in mouse liver during fasting [10] (unpublished, Kristensen et al.). Together with the present finding that p62 protein content decreased after 18 h of fasting indicating removal of p62, and hence increased autophagy this suggests induction of autophagy during fasting. This may be a fasting response providing nutrients as previously suggested [10]. Taken together, the current findings support that autophagy is a process induced during states of low nutrient availability, such as exercise and fasting and may function to increase the level of energy substrates or to remove damage cellular components after exercise, independent of the duration of the metabolic challenge. However, without the use of autophagy inhibitors, interpretations regarding autophagy must be made with caution and further experiments are needed to conclude on autophagy flux.

The present observation that lack of liver PGC-1α did not result in any changes in protein or phosphorylation level of UPR components in resting mice suggests that PGC-1α in the liver is not involved in the regulation of basal hepatic UPR. This is in contrast to a previous study reporting increased hepatic BiP protein in young whole-body PGC-1α mice [23]. However, global versus liver-specific PGC-1α knockout may explain this discrepancy. In addition, the finding that the fasting and exercise-induced changes in UPR markers observed in lox/lox mice also were present in LKO mice, apart from sXBP1 mRNA during exercise, indicates that the fasting-induced and exercise-induced regulation of UPR in the liver did not either require PGC-1α. This is supported by the observation that PERK phosphorylation and IRE1α protein content increased with exercise only in LKO mice, which may be related to the LKO mice being more challenged than lox/lox mice during the exercise bout.

The finding that the exercise-induced increase in the LC3II/LC3I ratio in the liver also occurred in the LKO mice indicates that PGC-1α is not required for the exercise-induced regulation of autophagy in mouse liver. This is different from the reported PGC-1α dependent exercise-induced autophagy in mouse skeletal muscle [5, 14, 37] suggesting tissue-specific regulation of autophagy. However, the higher LC3II/LC3I ratio observed in LKO than lox/lox mice within both resting groups may suggest that the basal level of autophagy in the liver is regulated by PGC-1α. It should however be noted that this genotype difference was absent in the fasting study, and therefore this warrants further investigations. The present finding that autophagy was regulated similarly in both genotypes during fasting supports that PGC-1α is not involved in regulation of autophagy during metabolic challenges in the liver.

The observation that liver-specific PGC-1α knockout did not result in altered exercise and fasting-induced regulation of UPR and autophagy is in line with the present finding that the classical exercise and fasting responses with decreased liver glycogen and increased hepatic triglyceride content were uncompromised in LKO mice. Previous studies have demonstrated that liver PGC-1α is involved in the expression of gluconeogenic proteins including PEPCK and G6Pase and enzymes in ketogenesis during fasting [9, 15, 35, 41]. However, the present observation that LKO mice increased PEPCK protein and plasma β-hydroxybutyrate in response to exercise and fasting similar to lox/lox mice suggests that liver PGC-1α was not required for these responses in the present study. This is in accordance with a previous study reporting that the exercise and fasting-induced increase in hepatic PEPCK and G6Pase was similar between wildtype and whole-body PGC-1α knockout mice [13]. It should be noted that LKO mice decreased glycogen earlier than lox/lox mice, indicating that these mice have been more challenged by the exercise bout than lox/lox mice. This was however not reflected in a more pronounced regulation of UPR and autophagy in LKO mice.

In conclusion, the present study provides evidence for exercise-induced activation of the three UPR pathways and regulation of autophagy in mouse liver independent of PGC-1α. Furthermore, fasting seems to decrease the capacity of specific UPR pathways as well as to regulate autophagy in the liver independent of PGC-1α. However, PGC-1α may be involved in regulating basal hepatic autophagy.
